# Family and job microsystems as mediators between social integration and depression among rural-to-urban migrant workers in China: does having sons make a difference?

**DOI:** 10.3389/fpubh.2024.1406451

**Published:** 2024-06-27

**Authors:** Guanghui Shen, Jiayi Tang, Juan Fang, Jiahui Huang, Yawen Zheng, Liujun Wu, Xudong Yang, Yu-Hsin Chen, Li Chen

**Affiliations:** ^1^Wenzhou Seventh People's Hospital, Wenzhou, China; ^2^School of Mental Health, Wenzhou Medical University, Wenzhou, China; ^3^Cixi Biomedical Research Institute, Wenzhou Medical University, Ningbo, China; ^4^Lishui Second People’s Hospital, Lishui, China; ^5^The Affiliated Wenzhou Kangning Hospital, Wenzhou Medical University, Wenzhou, Zhejiang, China

**Keywords:** social integration, depression, family happiness, job burnout, migrant workers, son preference, social-ecological systems

## Abstract

**Background:**

Rural-to-urban migrant workers are a vulnerable group at risk of developing depression. Based on the social-ecological systems theory, this study investigates the impact of the lack of social integration on depression, considering the mediating roles of migrant workers’ microsystems (family happiness and job burnout). Additionally, the study explores whether having sons influences these associations.

**Methods:**

The sample of 4,618 rural-to-urban migrant workers was obtained from the 2018 wave of the China Labor Force Dynamics Survey (CLDS). All the measures in the survey exhibited good reliability, including the Center for Epidemiological Research Depression Scale (CES-D), family happiness, job burnout, and social integration. The data were primarily analyzed using a structural equation model.

**Results:**

Social integration had a direct impact on depression among migrant workers. Additionally, it indirectly affected depression through the mediating roles of family happiness not job burnout. The moderating effect of having sons mainly occurred on the path from social integration to family happiness.

**Limitations:**

The cross-sectional design impeded the ability to draw causal inferences.

**Conclusion:**

This finding highlights the potential benefits of social integration and family happiness in promoting early prevention of depression among migrant workers. It indicates that the inclination toward having sons among migrant workers continues to impact their mental health.

## 1 Introduction

Rural-to-urban migrant workers in China is one of the largest mobile labor forces in the world, estimated to reach 0.29 billion by 2022 (1). This group often moves between their rural hometowns and temporary residences in the city due to the absence of permanent urban residency (‘hukou’) (2). As a result, many of them encounter difficult living conditions, poverty, low social status, uncertainty in self-identification, and a transient lifestyle, all of which are considered risk factors for experiencing depression (3–5).

In fact, the prevalence of depressive symptoms among migrant workers in China has reached 23.7%, indicating that nearly 60 million migrant workers suffer from depression (6). Recent studies focusing on social integration have offered new insights into the prevalence of depression among these workers (7). In China, urban and rural areas observed are two different systems that depend on each other, integrate with each other and complement each other (8). Due to the urban–rural difference and integration system, migrant workers face challenges in fully integrating into host cities, which makes them more vulnerable to mental health issues like depression, anxiety, and insomnia. Recent reports have highlighted the significance of social and urban isolation as a contributing factor to depression among migrant workers (9, 10). However, there is a lack of systematic investigations and discussions on the underlying mechanisms of urban isolation and depression in this population. Based on previous literature on migrants, the Social-Ecological System Theory (SEST) can serve as a suitable framework for exploring the potential mechanism that links the lack of social integration to depression (11). The SEST suggests that the social environment functions as a social ecosystem, where individual behavior and health are influenced by the interaction between humans and their environment. This interaction encompasses microsystems and macrosystems. The microsystem is the most influential level of the ecological systems and the macrosystem can impact an individual’s health through their microsystem (12, 13). Therefore, depression experienced by migrant workers may be influenced by the interaction of macrosystems and microsystems. In the context of rural-to-urban migrant workers in China, social integration is considered a core variable in the macrosystem due to the unique challenges they face, such as social isolation, cultural barriers, and limited access to social welfare (14, 15). Their migrant status often leads to family separation (16) and work-related stress (17), increasing their vulnerability to mental health issues like depression. In this context, family happiness and job burnout emerge as the main influencing factors in the microsystems.

Further, rural-to-urban workers in China poses a blend of traditional and modern cultural values due to their distinct migrant lifestyle. On the one hand, being born in rural areas, they are deeply influenced by the traditional culture that favors sons (18). On the other hand, working in urban cities exposes them to the modern culture of gender equality, which subtly impacts their beliefs (8, 19). Given this clash between traditional and modern cultural values, it is worthwhile to investigate the potential impact of having sons on the relationship between social integration and depression.

Accordingly, the present study aims to investigate the relationship between social integration (macrosystem) and depression (individual health) among rural-to-urban workers. It also explores the potential mediating effects of family happiness and job burnout (microsystem) and moderating role of having sons in this relationship.

### 1.1 Social integration and depression

Social integration refers to the extent to which individuals participate in a variety of social relationships, including engagement in social activities or relationships and a sense of communality and identification with one’s social roles (20, 21). Several studies have demonstrated that the lack of social integration can lead to mental problems like depression, loneliness, and insomnia (7, 22, 23). For example, a comprehensive review study, comprising 128 articles from 15 countries, clearly demonstrates that social isolation and loneliness are risk factors negatively impacting mental and physical health, including depression and cardiovascular health (24).

In China, there is a growing trend of rural-to-urban migrant workers who aspire to settle in cities and become permanent members of the host society (25). However, they still face various challenges such as institutional barriers, cultural obstacles, social welfare discrimination, and labor market segmentation, which hinder their complete integration into urban society (26). Based on the theory of SEST, social integration falls under the macrosystem and has a direct or indirect influence on individual’s mental health. Individuals with lower levels of social integration are more likely to experience a reduced sense of group belonging and meaning in life, increasing their vulnerability to depression (27). Therefore, rural-to-urban migrant workers, due to the barriers they encounter in socioeconomic integration in cities, are considered a vulnerable group at risk of developing depression. This paper presents the first hypothesis as follows:

*H1*: The social integration of migrant workers can negatively predict depression.

### 1.2 The mediating effects of family happiness and job burnout

According to the theory of SEST, the microsystem exerts the most significant influence on individual’s health within the ecological systems (28, 29). This study aims to investigate the impact of two specific microsystems: family happiness and job burnout.

From the perspective of the family microsystem, the strong positive correlation between family happiness and mental health among migrant workers has been extensively proved (30, 31). For instance, in a recent study by Shi et al. (32), a survey of 14,344 Chinese adults provided evidence supporting the idea that family happiness has protective effects on physical health and reduces the risk of depression. Similar correlations have also been found in the job microsystem. Job burnout is characterized by emotional exhaustion, depersonalization, and a reduced sense of work-related personal achievement (33). It has been identified as an important factor affecting individuals’ mental health (34, 35). For instance, in a pioneering study, Yang et al. (36) surveyed 1,595 male rural-to-urban migrant workers and discovered a positive association between higher work stress and the risk of probable mental disorders, such as sensitivity, anxiety, and depression.

**Figure 1:**
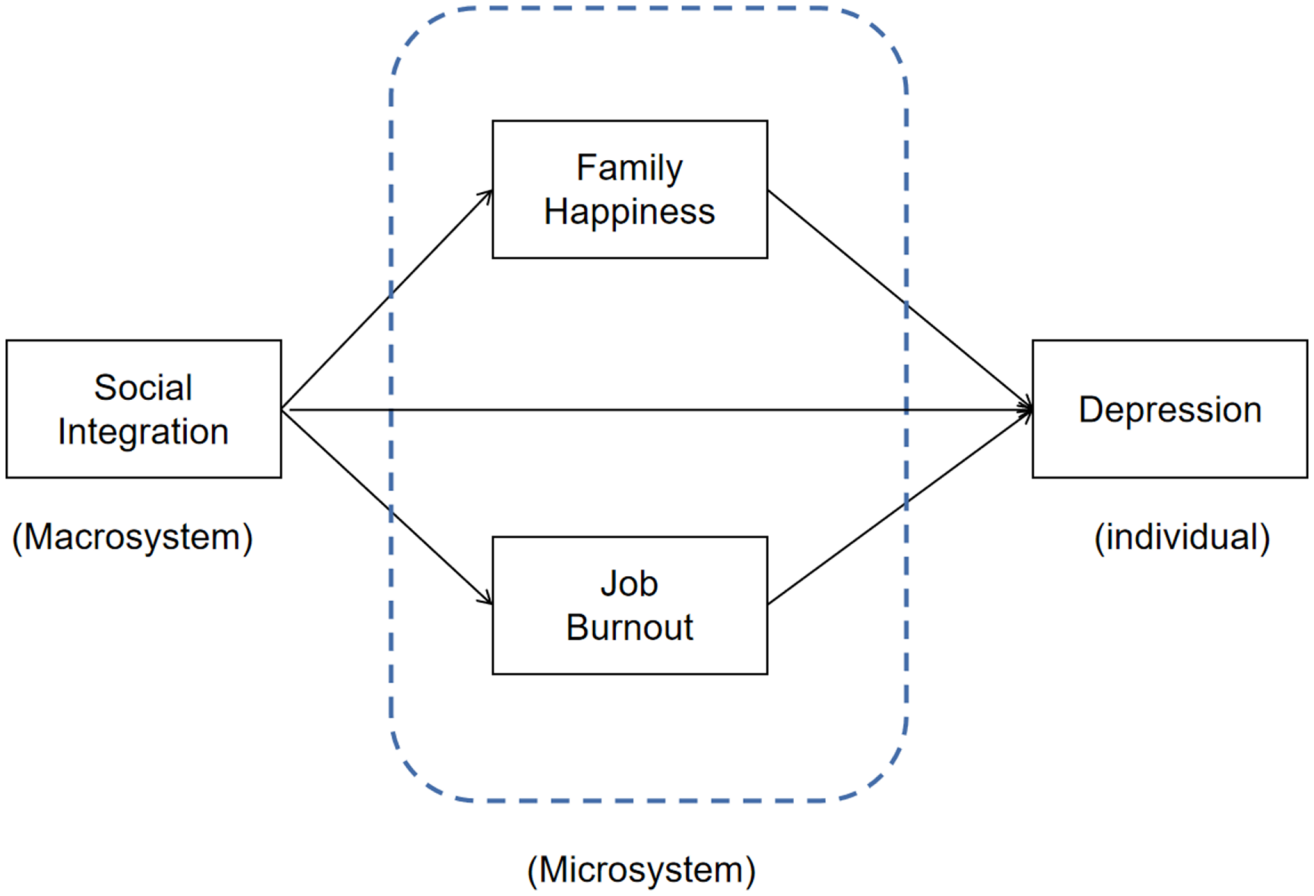
Conceptual framework.

Further, according to the theory of SEST, the macrosystem can have an influence on an individual’s microsystem (37, 38). Specifically, when there is a lack of social integration in the macrosystem, it creates a stressful environment that can result in negative effects in the microsystem. These negative effects may include job burnout and decreased family satisfaction (39). Therefore, it can be inferred that rural-to-urban migrant workers who have low levels of social integration in the macrosystem are more likely to experience high levels of job burnout and dissatisfaction within their family in the microsystem. Based on this understanding, the second hypothesis of this study is proposed (see Figure 1 for the conceptual framework):

*H2a*: Family happiness plays a mediating role between social integration and depression.

*H2b*: Job burnout plays a mediating role between social integration and depression.

### 1.3 The moderating effect of having sons

Son preference is a well-known gender bias that exists in Eastern cultural contexts (40, 41). In China, this bias is reinforced by the emphasis on the role of sons in religious family rites and their recognition as valid heirs to succession. Sons are considered permanent members of their family, as the family name can only be continued through sons (18). Moreover, sons are expected to provide economic supports for their parents in cases of aging, disability, or ill health (42). As a result, parents perceive sons as an investment or insurance for future and consider it crucial for a fulfilling life within a culture that endorses son preference. For example, a longitudinal study conducted in China involving 10,769 older adults revealed that a culture of son preference tends to be intensified in rural areas (43). Furthermore, the absence of care and attention from a son was found to contribute to a noticeable mental health disadvantage among Chinese adults, leading to feelings of depression, fear, loneliness, and desperation. Another influential study has empirically demonstrated that son preference significantly impacts the reproductive behavior of rural-to-urban migrant women in China (44). The study found that women who give birth to a daughter as their first child are more likely to have a second child compared to those whose first child is a son. In China, migrant workers who were born in rural areas are heavily influenced by the traditional culture of son preference (45). Their social status, sense of pride, and even family stability are closely tied to sons (46). Therefore, the absence of son is seen as a humiliation for migrant workers, which can further amplify the impact of insufficient social integration on their family happiness and mental well-being. Therefore, the third hypothesis of this study is proposed as follows:

*H3*: Having sons moderates the relationship between social integration and depression.

## 2 Methods

### 2.1 Data and sampling

The data used in this study was obtained from the 2018 China Labor Force Dynamics Survey (CLDS). The CLDS is a national longitudinal social survey targeted at the labor force in China and employs a multi-stage clustering and stratified probability proportional to size (PPS) sampling strategy conducted by Sun Yat-sen University (47). It is a project covering 28 provinces or cities in mainland (except for Hong Kong, Macao, Taiwan, Hainan, and Tibet). The study is conducted in accordance with the Declaration of Helsinki, and the protocol is approved by the Institutional Review Board of Sun Yat-sen University. More information on the design, sampling procedures, and methodology is documented elsewhere (48, 49).

The first round of CLDS was implemented in 2012 and followed up every 2 years. For the purpose of this study, data responses were retrieved from the 2018 CLDS dataset. The sample consisted of married migrant workers with rural area household registration. These individuals had engaged in one or more non-farm jobs for at least 3 months in the past year. Samples with missing or singular values were excluded, resulting in a final sample size of 4,618 participants.

### 2.2 Participants

Among the 4,618 migrant workers in the 2018 CLDS, 2277 (49.3%) were male and the average age was 45.77 ± 7.02 years. 2029 (43.9%) participants were from north China and 2,589 (56.1%) participants were from southern China, based on their registered permanent residence. 3,569 (77.3%) participants had at least one son, while 1,049 (22.7%) participants did not have sons.

### 2.3 Measurements

#### 2.3.1 Demographic variables

Demographic variables included age (continuous), gender (binary, 0 for female and 1 for male), Hukou location (binary, 0 for north China and 1 for south China) and children’s gender (binary, 0 for female and 1 for male) and numbers (continuous) using self-administered questions. Two questions were used to assess children’s gender and numbers: (1) *how many children do you have?* (2) *how many sons do you have?* The recoding principle for the number of sons is as follows: 0 = without sons; 1 = with sons (having one or more sons).

#### 2.3.2 Social integration

Social integration was measured using three questions (50): (1) *How familiar are you with your neighbors and other residents in this community?* (2) *Do you trust your neighbors and other residents in this community?* (3) *Do you, your neighbors, and other residents in this help each other?* All questions were answered on a five-point Likert scale from very little to very much. Higher scores indicate a greater degree of social integration. Cronbach’s α was 0.76 with the current samples.

#### 2.3.3 Family happiness

Family happiness was measured by three questions: (1) *Generally speaking, do you live a happy family life now?* (2) *Generally speaking, are you happy with your family living condition?* (3) *Generally speaking, are you happy with your family’s financial condition?* All questions were answered on a five-point Likert scale from unhappy to very happy, which means the levels of residents’ happiness increase as the number increases subjective happiness. Cronbach’s α was 0.80 with the current samples.

#### 2.3.4 Job burnout

Job burnout was measured by four items: (1) *Work makes me feel physically and mentally exhausted*. (2) *Working all day is stressful for me*. (3) *I’m less and less interested in this job*. All questions were answered on a five-point Likert scale from per day to none, and higher scores reflect lower job burnout. Cronbach’s *α* was 0.78 with the current samples.

#### 2.3.5 Depression

Depression was measured by the Center for Epidemiological Research Depression Scale (CES-D). The CES-D consists of 20 items. Each items were scored on a four-point Likert scale, from 0 (=<1 day) to 3 (=5–7 days). The higher the score, the higher level of depression (51). Cronbach’s *α* was 0.95 with the current samples.

### 2.4 Measurement tool verification

**Figure 2:**
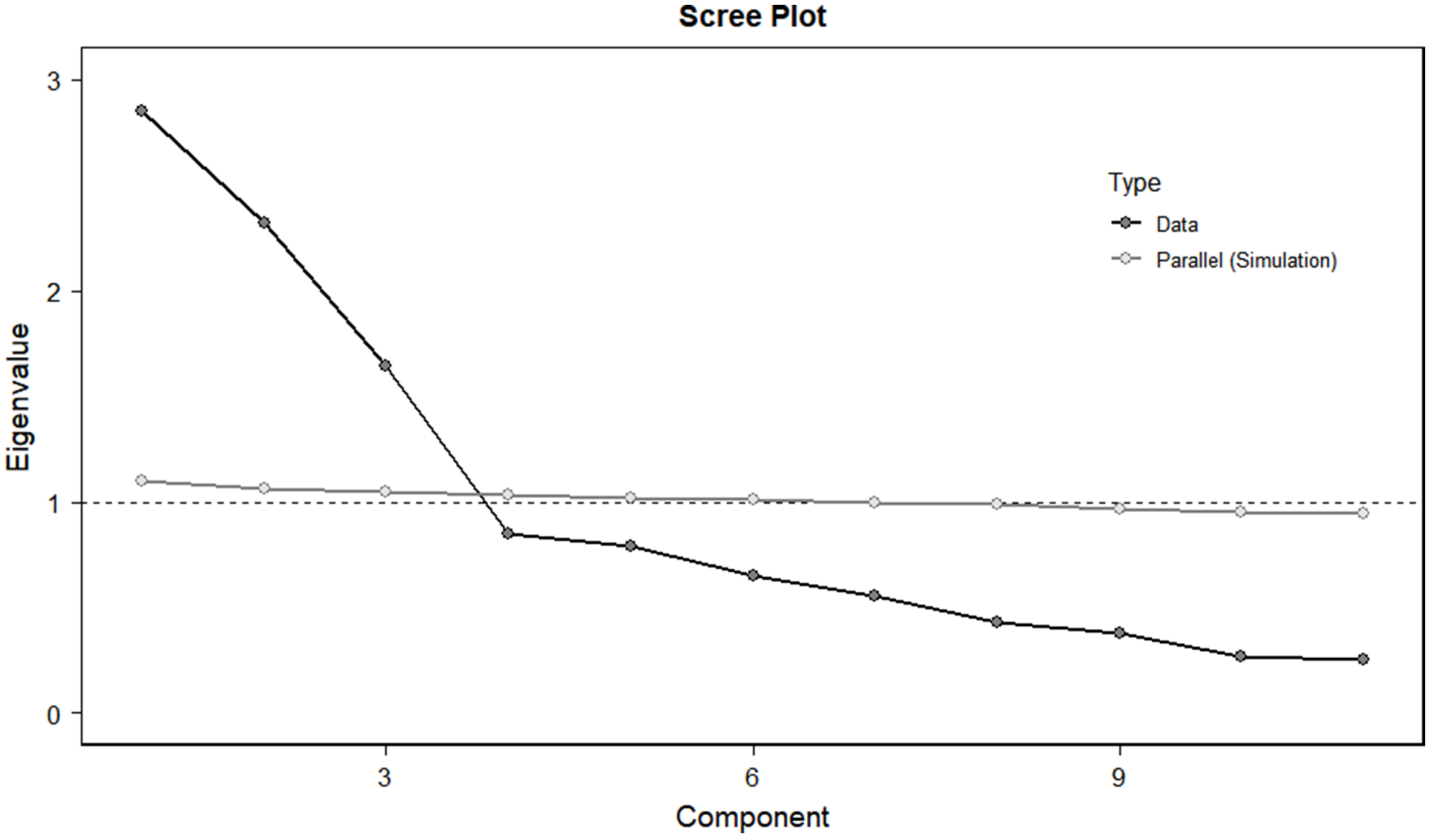
Scree plot indicating an optimal 3-factor solution for the measurement items.

The study utilized a self-compiled questionnaire to measure social integration, family happiness, and job burnout. To investigate the psychometric properties of the measurement tool, exploratory factor analysis (EFA) and confirmatory factor analysis (CFA) were conducted. The 2016 CLDS data was used for EFA to identify the factorial structure of the items. Subsequently, the factorial structure was further validated using the 2018 CLDS data through the adequacy of the best measurement model in CFA. The eligibility of the data for exploratory factor analysis was assessed using the Kaiser–Meyer–Olkin (KMO) Measure of Sampling Adequacy and Bartlett’s Test of Sphericity. The result was appropriate for factor analysis [KMO = 0.722; *χ*^2^_(45)_ = 42692.77, *p* < 0.001]. Principal component analysis with orthogonal (varimax) rotation are used to extract factors, extract criteria are as follows: (1) The factor loading on the factor is higher than 0.50. (2) No factor loadings ≥0.5 in more than one factor. (3) Factors with eigenvalues greater than 1. (4) Each factor contains at least 3 items. Finally, produced three factor, which accounted for 65.52% of the variance, and all items had a loading ≥0.5 (see Table 1 and Figure 2). Factor 1, job burnout, explained 20.17% of the variance and it included the items 1, 2, and 3; Factor 2, family happiness, explained 23.05% of the variance and it included the items 4, 5 and 6; Factor 3, social integration, explained 22.30% of the variance and it was comprised of items 7, 8, and 9. Table 1 presents the item loadings on the three factors. Then CFA was used to evaluate the validity of three-factor structure in the 2018 data. The results demonstrated an excellent fit of model: *χ*^2^ = 548.95, *df* = 41, CFI = 0.968, NFI = 0.966, RMSEA = 0.05, all observed variables were significantly loaded on the corresponding latent constructs at the *p* < 0.05 level (52).

**Table 1:** Forced 3-factor solution by principal components analysis and orthogonal varimax rotation for the measurement items.

Measurement items	Factor		
	Job burnout	Family happiness	Social integration
1. Work makes me feel physically and mentally exhausted	0.854		
2. Working all day is stressful for me	0.868		
3. I’m less and less interested in this job?	0.700		
4. Generally speaking, do you live a happy family life now?		0.864	
5. Generally speaking, are you happy with your family living condition?		0.900	
6. Generally speaking, are you happy with your family’s financial condition?		0.783	
7. How familiar are you with your neighbors and other residents in this community?			0.828
8. Do you trust your neighbors and other residents in this community?			0.838
9. Do you, your neighbors, and other residents in this help each other?			0.798

### 2.5 Statistic analysis

Statistical analyses were performed using SPSS 20.0 for Windows and *R* 4.1.2. Pearson’s correlation analyses were carried out to preliminarily describe the correlations among social integration, family happiness, job burnout, depression, and demographic variables. To test the mediation effect, the *lavaan* package (R package; version 0.6–9) was used to create the structural equation model (SEM). Firstly, the SEM was constructed in whole-sample and the mediation effects were estimated by bootstrap. After that, multi-group SEM was constructed to test the potential moderator of having sons. That is, whether the mediation model shows significant differences between the groups with sons and without sons. Three basic steps were followed to make multi-group SEM. Firstly, all parameters were freely estimated across two samples (with sons and without sons) and made a free estimation model (Model 1). Then, constrain the factor loading of latent variables to be equal in different samples and build a factor-invariant model (Model 2). Thirdly, construct the path coefficients to be equal across samples and establish an equal factor correlation model (Model 3) (53).

Model fit is assessed in multiple ways. First, use the *χ*^2^ test to assess fit. According to previous research, *χ*^2^/df values less than 5 are a criterion for a great fit (54). However, since the *χ*^2^ test depends on the sample size, the large sample size in study may lead to failure. Therefore, the goodness-of-fit indices, specifically, the Comparative Fit Index (CFI) and Normal of Fit Index (NFI) and the Root Mean Square Approximation Error (RMSEA), were also assessed. CFI and NFI with values greater than 0.90 indicate great fit, and RMSEA values less than 0.07 indicate good fit (55).

## 3 Results

### 3.1 Preliminary analysis

The means and standard deviations of study variables are shown in Table 2. Table 2 presents Pearson’s correlations among the study variables. As expected, depression was significantly correlated with social integration (*r* = −0.09, *p* < 0.001), family happiness (*r* = −0.32, *p* < 0.001) and job burnout (*r* = 0.33, *p* < 0.001). Social integration was significantly positively correlated with family happiness (*r* = 0.10, *p* < 0.001). Interestingly, social integration showed no significant relationship with job burnout (*r* = −0.02, *p* = 0.081), and having sons was significantly positively correlated with social integration. Independent sample *t-*test revealed that parents with at least one son are significantly more socially integrated than parents without sons (with sons: 17.60 ± 2.88; without sons: 17.07 ± 2.97; *t* = 5.06, *p* < 0.001).

**Table 2:** Pearson correlations and descriptive statistics for the study variables.

	1	2	3	4	5	6	7
1. Gender	1						
2. The number of sons	0.03^*^	1					
3. Age	−0.01	0.15^***^	1				
4. Social integration	−0.09^***^	0.09^***^	0.22^***^	1			
5. Depression	0.06^***^	0.02	0.03^*^	−0.09^***^	1		
6. Job burnout	−0.01	0.07^***^	0.04^**^	−0.02	0.33^***^	1	
7. Family happiness	0.001	−0.01	−0.05^**^	0.10^***^	−0.32^***^	−0.23^***^	1
Mean	(–)	(–)	45.77	17.47	27.21	10.80	10.73
*SD*	(–)	(–)	7.02	2.91	8.64	3.94	2.35

^*^p < 0.05, ^**^p < 0.01, ^***^p < 0.001.

### 3.2 The mediators of family happiness and job burnout

**Figure 3:**
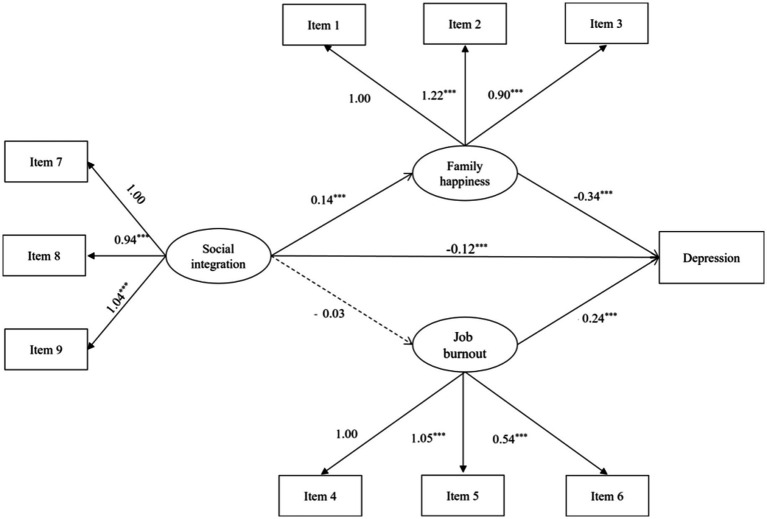
Path models for parallel multiple mediator. ***p < 0.001.

Structural equation modeling was used to examine the mediating role of family happiness and job burnout between social integration and depression. The mediation model obtained optimal fit indices (*χ*^2^ = 919.44, *df* = 45, CFI = 0.946, NFI = 0.943, RMSEA = 0.065). Figure 3 presents the standardized coefficient of the parallel mediation model. After controlling for age and gender, social integration (*β* = −0.12, *p* < 0.001) and family happiness (*β* = −0.35, *p* < 0.001) were negatively predicted depression level, but job burnout (*β* = 0.24, *p* < 0.001) was positively predicted depression level.

The mediating effects of family happiness and job burnout estimated by bootstrap are shown in Table 3. As the bootstrapped 95% confidence interval around the standardized indirect effect did not include zero, social integration had a significant direct effect on depression level (Effect = −0.12, *p* < 0.001, 95% bootstrap CI [−0.18, −0.06]). Meanwhile, social integration had a significant indirect effect on depression level via family happiness (Effect = − 0.04, *p* < 0.001, 95% bootstrap CI [−0.06, −0.02]). Direct and indirect effects accounted for 71 and 29% of the total effect, respectively. However, job burnout did not play a mediating role in the relationship between social integration and depression (Effect = −0.01, *p* = 0.439, 95% bootstrap CI [−0.01, 0.01]).

**Table 3:** The bootstrap confidence interval and effect size of the mediation model.

Defined	Effect	S.E.	t	p	Bootstrapping *CI*
Social integration → Job burnout → Depression	−0.01	0.01	−0.77	0.439	[−0.01, 0.01]
Social integration→ Family happiness → Depression	−0.04	0.01	−5.76	<0.001	[−0.06, −0.02]
Social integration → Depression	−0.12	0.03	−4.31	<0.001	[−0.18, −0.06]
Total Effect	−0.17	0.03	−5.36	<0.001	[−0.23, −0.11]

### 3.3 Group differences in having sons

Multi-group analysis in SEM was used to test group differences in having sons. Fitting free-estimation model (model 1), factor-invariant model (model 2), and equal-factor correlation model (model 3), separately. The model fit index indicates that the above three models fit well (CFIs >0.90, NFIs >0.90, RMSEAs <0.07, see Table 4). However, the result of the model comparison showed that model 2 and model 3 fits were significantly different from model 1. Specifically, the constraints model 2 and model 3 added on model 1 significantly reduced the model according to Chi-square tests (model 2: Δ*χ*^2^ = 33.21, *p* < 0.001; model 3: Δ*χ*^2^ = 40.84, *p* < 0.001), as shown in Table 4. The above results reveal that the two groups follow our theoretical framework, but the path coefficients and residuals of the model are significantly different in the two groups. Further, the critical ratios (CR) was used to compare the path coefficients, as shown in Table 5. The structural path from social integration to family happiness was significantly different across the two groups (with sons: *β* = 0.16, *p* < 0.001; without sons: *β* = 0.07, *p* = 0.045; CR = 2.67, *p* < 0.001). There were no significant differences seen in other structural paths. Those results demonstrated whether having sons or not will have a moderating effect on the theoretical framework of the parallel mediation model proposed in this study, and this moderating effect mainly occurred on the path from social integration to family happiness. Specifically, social integration had a more significant effect on the family happiness of migrant workers with sons.

**Table 4:** Model comparison of structural model for full sample.

Fit indexes	*χ* ^2^	Df	Δ*χ*^2^	p	RMSEA	CFI	NFI
Free-estimation model	1083.78	112	(–)	(–)	0.061	0.942	0.935
Factor-invariant model	1113.58	119	29.80	<0.001	0.060	0.940	0.934
Equal-factor correlation model	1119.41	124	35.63	<0.001	0.059	0.940	0.933

**Table 5:** Multi-group critical ratio analysis.

Path coefficients	Without Sons	Sons	Critical ratio
Social integration → Depression	−0.09^*^	−0.13^***^	1.44
Job burnout→ Depression	0.27^***^	0.24^***^	1.13
Family happiness→ Depression	−0.34^***^	−0.35^***^	1.03
Social integration → Job burnout	−0.06	−0.04	1.50
Social integration → Family happiness	0.07^*^	0.17^***^	2.43^***^

^*^p < 0.05, ^***^p < 0.001.

## 4 Discussion

As a significant public health concern, the depression of rural–urban migrant workers has attracted increased attention globally. This study contributes to existing knowledge by exploring the prevalence of depression among Chinese migrant workers from the perspective of social integration, using the SEST as a framework. The study also investigates the mediating role of family happiness and job burnout in the relationship between social integration and depression, highlighting the importance of the SEST framework in understanding the pathways linking social integration and depression. Additionally, the study evaluates the moderating role of having sons.

### 4.1 Social integration and depression

The initial finding of the study revealed a significant association between social integration of migrant workers and depression. It was observed that migrant workers who had lower levels of social integration experienced depression more frequently. This finding supports the H1 and aligns with the results of previous studies (38, 56, 57). In a recent systematic review examining the relationship between social relations and depression, it has been found that social integration, which includes factors such as social support and having a diverse network of social connections, has a significant protective effect against depression (58). Additionally, the social ranking theory suggests that social status plays a crucial role in human social interactions, making it challenging for individuals from lower social classes to integrate into higher social class groups. Consequently, the lower social rank of migrant workers in urban areas exacerbates social conflicts and impedes their integration into cities, due to the existing social, economic, and cultural disparities. According to the SEST, migrant workers with lower social rank often face challenges in integrating into the macrosystem. This difficulty in social integration may increase the risk of individual depression in the microsystem among migrant workers.

### 4.2 Family happiness and job burnout as the mediators

The second important finding of this study was that social integration indirectly influenced depression through its impact on family happiness, rather than job burnout. Family happiness acted as a partial mediator in the relationship between social integration and depression, supporting H2a of the second hypothesis. The study revealed a significant positive correlation between social integration and family happiness. Additionally, a moderate negative correlation was observed between family happiness and depression, indicating that higher levels of family happiness were associated with lower levels of depression, consistent with previous research (30, 38, 59). The process of rural-to-urban migration involves separation from family members and disruption of family life (60). Migrant workers who experience higher levels of social integration in their host cities have more opportunities to maintain family connections, receive social support, and navigate the challenges of balancing work and family responsibilities (61), which contribute to greater family well-being and, subsequently, lower levels of depression. However, job burnout did not act as a mediator between social integration and depression. This non-significant result may be attributed to the unique employment experience and challenged faced by migrant workers. Migrant workers often experience high levels of work stress, including long working hours, low wages and limited job security (31, 62). These pervasive objective stressors obscure the potential impact of social integration on job burnout in this group. Moreover, in Chinese culture, family is often considered the primary source of support and well-being (63). Social integration plays a more significant role in facilitating family well-being than in reducing job burnout, given the cultural emphasis on family relationships and the challenges of maintaining family connections during the migration process.

According to the SEST, the relationship between social integration and depression among rural-to-urban workers is influenced by the microsystem, particularly the family microsystem. The family microsystem, being the most stable and innermost system for each individual, significantly impacts individual development. Migrant workers who lack social integration may experience family conflict or dissatisfaction, which can subsequently increase their likelihood of experiencing depression. Conversely, the job microsystem for migrant workers is characterized by a wide range of uncertain and unstable factors. In other words, while social integration can easily affect the family microsystem, which is closely associated with migrant workers, improving work performance and reducing job burnout in the job microsystem is challenging due to temporary immigration status. These findings highlight the significance of family happiness for the mental well-being of migrant workers during their process of social integration.

### 4.3 Son preference as the moderator

The study’s third important finding reveals the significant moderating effect of having sons on the relationship between social integration and depression among rural-to-urban migrant workers in China. This finding supports the H3 and provides new evidence for the prevalence of son preference in China. Specifically, the social integration of the group with sons has a stronger impact on family happiness compared to the group without sons. This finding can be explained by the social and cultural factors that underlie son preference in China. From a sociocultural perspective, migrant workers who have sons experience greater social inclusion and support within their communities. In Chinese society, having sons is associated with higher social status and stronger social networks (64). Research by Wang et al. (65) demonstrates that this increased social integration buffers against the negative effects of migration-related stressors on mental health outcomes. The social capital and resources provided by sons help migrant workers navigate the challenges of adapting to urban life and maintain social connections. Furthermore, in a society that places a high value on male offspring, having sons contributes to a sense of pride, accomplishment, and continuity of the family lineage (64). This psychological well-being enhances the positive effects of social integration on mental health. As a result, migrant workers with sons are better equipped to navigate the challenges of adapting to urban life and maintain their mental health and well-being.

### 4.4 Limitations and future research

Our study had certain limitations that should not be overlooked, and it provides ideas for future research. Firstly, it is important to note that our study was based on a cross-sectional design, which prevents us from establishing a valid cause-and-effect relationship between social integration and depression. In the mediation model proposed in this study, it appears that social integration plays a role in enhancing family happiness, which in turn contributes to a decrease in depression. However, it is plausible that family happiness could be a consequence of depression rather than a cause. To establish causal relationships among these variables, it is imperative for future studies to utilize longitudinal data or panel data. Secondly, data from 2018 CLDS is a second-hand survey data, which is inevitably limited in terms of variable selection. Thirdly, it is important to acknowledge that although the CES-D scale is commonly used in epidemiological studies of depression, it is a survey tool and does not diagnose depressive disorders. Additionally, this study requires migrant workers to have been engaged in non-agricultural work for at least 3 months in the past year, which may bring some limitations to the generalizability of our findings. Future research should consider including migrant workers with different job categories to gain a more comprehensive understanding of factors affecting their mental health.

## 5 Implication and conclusion

The findings from this study have significant implications for current practice and future research. Firstly, based on the relationship found between social integration and depression among migrant workers with rural-to-urban experience, it is important for the Chinese government and society management department to pay more attention to the growing concern of depression among them. This can be achieved by implementing effective interventions. Secondly, considering that experiencing low levels of family happiness and high levels of job burnout are associated with a higher risk of depression, it is necessary to proactively provide risk assessment and mental health intervention strategies for migrant workers. Thirdly, although son preference may be considered outdated in terms of gender equality, it is crucial to recognize that sons can act as a protective factor for social integration and against depression.

In conclusion, this study provides empirical evidence for the relationship between social integration and depression in Chinese rural–urban migrant workers. Moreover, the level of social integration directly influences depression, or indirectly through the mediating role of family happiness. Further, the buffering effect of son preference is significant and remarkable.

## Data availability statement

The original contributions presented in the study are included in the article/supplementary material, further inquiries can be directed to the corresponding authors.

## Ethics statement

The studies involving humans were approved by the Institutional Review Board of Sun Yat-sen University. The studies were conducted in accordance with the local legislation and institutional requirements. The participants provided their written informed consent to participate in this study.

## Author contributions

GS: Writing – original draft, Methodology. JT: Writing – original draft, Conceptualization. JF: Investigation, Writing – original draft. JH: Investigation, Writing – original draft. YZ: Investigation, Writing – original draft. LW: Data curation, Writing – original draft. XY: Data curation, Writing – original draft. Y-HC: Writing – review & editing. LC: Writing – review & editing.
